# Glycocalyx Sensing with a Mathematical Model of Acoustic Shear Wave Biosensor

**DOI:** 10.3390/bioengineering9090462

**Published:** 2022-09-10

**Authors:** Varvara Turova, Andrey Kovtanyuk, Oleg Pykhteev, Irina Sidorenko, Renée Lampe

**Affiliations:** 1 Klinikum Rechts der Isar, Technische Universität München, Ismaninger Str. 22, 81675 München, Germany; 2 Fakultät für Mathematik, Technische Universität München, Boltzmannstr. 3, 85747 Garching bei München, Germany; 3 Interhyp AG, Domagkstr. 34, 80807 München, Germany

**Keywords:** acoustic sensor, shear waves, endothelial glycocalyx, preterm infants

## Abstract

The article deals with an idea of exploiting an acoustic shear wave biosensor for investigating the glycocalyx, a polysaccharide polymer molecule layer on the endothelium of blood vessels that, according to recent studies, plays an important role in protecting against diseases. To test this idea, a mathematical model of an acoustic shear wave sensor and corresponding software developed earlier for proteomic applications are used. In this case, the glycocalyx is treated as a layer homogenized over the thin polymer “villi”. Its material characteristics depend on the density, thickness, and length of the villi and on the viscous properties of the surrounding liquid (blood plasma). It is proved that the model used has a good sensitivity to the above parameters of the villi and blood plasma. Numerical experiments performed using real data collected retrospectively from premature infants show that the use of acoustic shear wave sensors may be a promising approach to investigate properties of glycocalyx-like structures and their role in prematurity.

## 1. Introduction

The endothelial glycocalyx (GL) is a gel-like layer composed of polysaccharide molecules that coats the luminal surface of blood vessels. Recent studies suggest that endothelial GL may help protect the vascular wall from disease [1]. The vasoprotective role of glycocalyx is known to be especially important in cerebral capillaries [2]. The cerebrovascular GL has been sparsely studied, but is of great interest because of its potential role in cerebrovascular disease. There is also evidence of glycocalyx importance in critically ill children, especially in premature infants [3]. Mathematical modeling and simulation of GL can aid in understanding the role of GL in proper endothelial function and consequences of its degradation.

There exist mathematical models that treat GL as a porous medium [4,5]. In [5], e.g., GL is modeled as a medium of variable and adaptive porosity. Some other works (see, e.g., [6]) use molecular dynamic simulation methods to mimic the dynamics of the GL and its components. In [7], the endothelial cells are modeled as a nearly incompressible hyperelastic neo-Hookean material. Different approximations of GL structure were investigated in [8]. It was demonstrated that only one model showed consistency in treating GL cells as a homogeneous elastic medium, namely, the brush model.

There are also some experimental works dealing with chemical fabrication of glycocalyx-mimetic surfaces (see, e.g, [9,10]). Such artificial structures can be used for a direct investigation of glycocalyx-like substances and their interaction with blood flow. For research purposes, acoustic shear wave sensors [11] whose high sensitivity is achieved due to the usage of shear horizontally polarized guided waves (Love waves), being surface waves with horizontal motion which is perpendicular to the direction of wave travelling, seem to be a good tool. Acoustic shear wave biosensors utilize surface acoustic waves excited in a piezoelectric substrate to detect and quantify proteins and cells in biological solutions (see, e.g., [12]). In the articles [13,14,15], a mathematical model of an acoustic biosensor has been developed and its adequacy and good performance in detecting small amounts of cells and molecules in biological solutions have been demonstrated.

The purpose of this article is to show that the mathematical model of the biosensor proposed in [13,14,15] can be used for sensing the properties of GL-like structures. The visualization and quantification of endothelial GL is a difficult problem. There are some experimental works on the usage of such imaging techniques (see [16] for a review) as transmission electron microscopy, intravital microscopy, micro-particle image velocimetry, confocal laser scanning microscopy, two-photon laser scanning microscopy, orthogonal polarization spectral imaging and sidestream dark field/oblique imaging for the analysis of GL. Most of the methods evaluate visual characteristics of GL. In this paper, the sensitivity of the acoustic biosensor’s model to changes in both geometric and material properties of structures mimicking the glycocalyx, including the properties of the surrounding fluid (blood plasma) will be demonstrated. Model simulations, using real clinical data of premature infants, will be presented.

## 2. Materials and Methods

### 2.1. Mathematical Model of Acoustic Wave Biosensor

**Figure 1:**
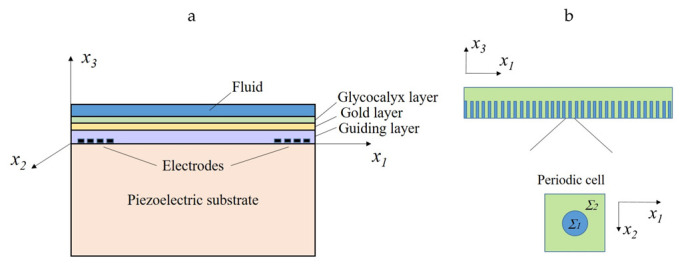
(**a**): Cross-section of a biosensor with the plane $x_{2}=0$; (**b**) Periodic structure of glycocalyx-like layer.

A detailed description of the mathematical model of an acoustic wave biosensor is presented in [13,14,15]. Here, we give a brief outline of the biosensor model. The operating principle of the biosensor is based on the piezoelectric excitation of acoustic shear waves using electrodes deposited on the left side of the surface of a plate specially cut from an α-quartz crystal (see Figure 1). The surface nature of acoustic waves is achieved due to the presence of a guiding layer made of silicon dioxide (SiO${}_{2}$) on the surface of the plate. The choice of materials must ensure that the wave velocity in the guiding layer is less than that in the substrate so that the waves will be transferred into the guiding layer. Acoustic waves propagate along the surface of the guiding layer (along the $x_{1}$ axis in Figure 1a). The wave propagation velocity is measured using a second array of electrodes located on the right side of the plate. The biosensor is modeled as a multi-layered structure with the bottom layer made from an ST-cut of piezoelectric alpha-quarz, a thin SiO${}_{2}$-guiding layer, a shielding gold layer, a bristle (villus) layer, and a fluid layer. An individual villus mimics one polymer molecule. It is supposed that the villus layer has a periodic structure (see Figure 1b): the diameter of villi is very small and their number is very large. The parameter $\theta =\Sigma_{1}/\Sigma_{2}$ in Figure 1b reflects the relation between the villus diameter and the distance between the villi.

The governing equations for the piezoelectric substrate are:

(1) $$\rho^{s}u_{i\;tt}-C_{ijkl}\frac{\partial^{2}u_{l}}{\partial x_{j}\partial x_{k}}-e_{kij}\frac{\partial^{2}\varphi }{\partial x_{k}\partial x_{j}}=0,$$

(2) $$\epsilon_{ij}\frac{\partial^{2}\varphi }{\partial x_{i}\partial x_{j}}+e_{ikl}\frac{\partial^{2}u_{l}}{\partial x_{i}\partial x_{k}}=0.$$

Here, i,j,k,l=1,2,3, $\rho^{s}$ is the density of the substrate material, $u_{i}$ is the displacement in $x_{i}$ direction, $C_{ijkl},\;e_{kij}$, and $\epsilon_{ij}$ are the elastic stiffness tensor, the stress piezoelectric tensor, and the material dielectric tensor, respectively, and φ is the electric potential. In the case of small deformations, the following material laws hold:

$$\sigma_{ij}=C_{ijkl}\varepsilon_{kl}-e_{kij}E_{k},$$

$$D_{i}=\varepsilon_{ij}E_{j}+e_{ikl}\varepsilon_{kl}.$$

Here, $\sigma_{ij}$ and $\varepsilon_{kl}$ are the stress and strain tensors, $D_{i}$ and $E_{i}=\partial \varphi /\partial x_{i}$ are the electric displacement and electric field.

The equation for the isotropic gold and guiding layers is:

(3) $$\rho^{g}u_{i\;tt}-C_{ijkl}\frac{\partial^{2}u_{l}}{\partial x_{j}\partial x_{k}}=0,\;i,\;j,\;k,\;l=1,\;2,\;3,$$

(4) $$\sigma_{ij}=C_{ijkl}\varepsilon_{kl}=\lambda \delta_{ij}\varepsilon_{kk}+2\mu \varepsilon_{ij},\;\mu =\frac{E}{2(1+\nu )},\;\lambda =\frac{E}{\left(1+\nu )(1-2\nu \right)},$$

where $\rho^{g}$ is the density, *E* is the Young’s module and ν the Poisson’s ratio of the corresponding layer material. Here, no electric field is present because of gold conductivity and low electric permeability coefficient of SiO${}_{2}$.

The fluid layer is described by the Navier-Stokes and mass conservation equations as follows:

(5) $$\rho^{f}v_{i\;t}-\eta \nabla v_{i}-\left(\zeta +\frac{\eta }{3}\right)\frac{\partial }{\partial x_{i}}div\vec{v}+\frac{\partial }{\partial x_{i}}p=0,$$

(6) $$\rho_{t}^{f}+\frac{\partial }{\partial x_{i}}\left(\rho^{f}v_{i}\right)=0.$$

Here, $\rho^{f}$ is the fluid density, η and ζ are the dynamic and volume viscosities of the fluid, $v_{i}$ are the $x_{i}$-velocity components, and *p* is the pressure.

Assuming the weakly compressibility of the fluid and using the substitutive relation

$$\rho^{f}\left(p\right)\approx \rho_{0}^{f}+\frac{\partial \rho^{f}}{\partial p}{|}_{entr}\left(p-p_{0}\right),$$

where $\rho_{0}^{f}=\rho^{f}\left(p_{0}\right)$ and $\partial \rho^{f}/\partial p{|}_{entr}$ is the density change under a constant entropy, the following equations for the fluid are obtained:

(7) $$\rho_{0}^{f}v_{i\;t}-\eta \nabla v_{i}-\left(\zeta +\frac{\eta }{3}\right)\frac{\partial }{\partial x_{i}}\mathrm{div}\vec{v}+\frac{\partial }{\partial x_{i}}p=0,$$

(8) $$\gamma p_{t}+\mathrm{div}\vec{v}=0.$$

Here, $\gamma =\frac{1}{\rho_{0}}\frac{\partial \rho^{f}}{\partial p}{|}_{entr}$ is the compressibility of the fluid.

For the bristle (glycocalyx) layer, the following equation has been obtained using a special homogenization technique developed in [13]:

(9) $$\rho^{GL}u_{i\;tt}-C_{ijkl}^{GL}\frac{\partial^{2}u_{l}}{\partial x_{j}\partial x_{k}}-P_{ijkl}^{GL}\frac{\partial^{2}u_{l\;t}}{\partial x_{j}\partial x_{k}}=0.$$

Here, the density $\rho^{GL}$ is a weighted combination of the density of the fluid and the density of the glycocalyx, the tensor $C^{GL}$ represents the elastic stresses of the homogenized structure, and the tensor $P^{GL}$ stands for viscous damping originated from the fluid part of the bristle structure. Both tensors can be computed with the Finite Element method using analytic representation of solutions of the so-called cell equation arising in homogenization theory.

The above equations are fully coupled using the following interface conditions.

1. On the interfaces between two solid layers, the continuity of the displacements and the equilibrium of normal pressures must hold.
2. The electric displacement and the tangent component of the electric field must be zero on the interface between the piezoelectric substrate and the guiding layer.
3. The equilibrium of the pressures and a no-slip condition is required on the interface between the fluid and homogenized bristle structure (glycocalyx).

### 2.2. Computer Program for the Calculation of the Acoustic Wave Velocity

The method of dispersion relations allowing the computation of the acoustic wave velocity depending on the wave frequency was elaborated and implemented in the form of user-friendly software [15,17]. In contrast to acoustic waves in bulk materials, the wave velocity in laminate structures depends on the frequency because of the interaction between the layers with different acoustic properties. Therefore, one can speak about dispersion relations that express the connection between the velocity and the frequency of acoustic waves. The algorithm is based on the construction of travelling wave solutions of equations describing deformations in the layers. The wave velocity is computed from the fitting of mechanical conditions on the interfaces between the layers. These conditions express the continuity of the displacement field and the pressure equilibrium for each pair of the layers. Feasible wave velocities are the roots of a nonnegative real function (fitting function) which measures the inconsistency in the interface conditions.

The details of the algorithm for computing acoustic shear wave velocity are given in [17]. Here we present a sketch of the method only. One looks for the acoustic shear waves propagating in $x_{1}$-direction and describing plain waves solutions in the form:

(10) $$u_{i}\left(x_{1},x_{3}\right)=a_{1i}\left(x_{3}\right)\cos\left(kx_{1}-\omega t\right)+a_{2i}\left(x_{3}\right)\sin\left(kx_{1}-\omega t\right),$$

(11) $$v_{i}\left(x_{1},x_{3}\right)=a_{3i}\left(x_{3}\right)\cos\left(kx_{1}-\omega t\right)+a_{4i}\left(x_{3}\right)\sin\left(kx_{1}-\omega t\right),$$

(12) $$\varphi \left(x_{1},x_{3}\right)=b_{1}\left(x_{3}\right)\cos\left(kx_{1}-\omega t\right)+b_{2}\left(x_{3}\right)\sin\left(kx_{1}-\omega t\right),$$

where *k* is the wave number and ω is the circular frequency related to the operating frequency *f* of the sensor as ω=2πf. Substituting (10)–(12) in the Equations (1), (2), (3), (7), (8), and (9) and equating the coefficients on cos and sin, one obtains a system of ordinary differential equations for the coefficients $a_{li}$, l=1,⋯,4, and $b_{s}$, s=1,2, in each layer. These systems are solved for every layer to obtain solutions in the following form:

(13) $${\vec{a}}_{l}\left(x_{3}\right)=\left(a_{l1},a_{l2},a_{l3}\right)=\sum_{j}A_{l}^{j}{\vec{h}}_{l}^{j}\exp\left(\lambda_{l}^{j}kx_{3}\right),\;b_{s}\left(x_{3}\right)=\sum_{j}B_{s}^{j}{\vec{g}}_{s}^{j}\exp\left(\lambda_{s}^{j}kx_{3}\right).$$

Here, $A_{l}^{j}$ and $B_{s}^{j}$ are unknown coefficients, ${\vec{h}}_{l}^{j}$ and ${\vec{g}}_{s}^{j}$ are eigenvectors, and $\lambda_{l}^{j}$ and $\lambda_{s}^{j}$ are eigenvalues of the matrix of the corresponding system of differential equations.

**Figure 2:**
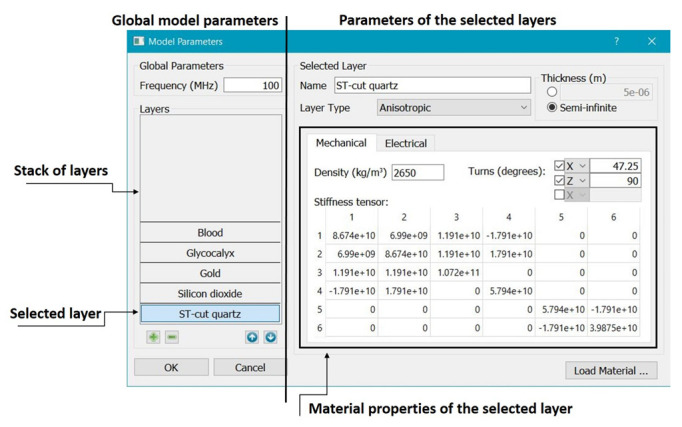
Snapshot of the user interface computer program for calculating velocity of acoustic waves.

The travelling wave solutions in the whole multi-layered structure are computed via the substitution of the functions (13) (with the substituted expressions for $a_{li}$ and $b_{s}$) into the interface conditions. As a result, one arrives at a homogeneous system of algebraic equations for unknown coefficients $A_{l}^{j}$, $B_{s}^{j}$. Denoting the unknown wave velocity by V=ω/k and fixing the circular frequency ω, one can consider the matrix *G* of this system as a function of *V*. Finally, one arrives at the feasibility condition for the existence of a nontrivial solution in the form: $det|{\bar{G}}^{T}\left(V\right)G\left(V\right)|=0.$ A snapshot of the user interface of the computer program for solving this equation is shown in Figure 2. To obtain the velocity of waves and displacements in different layers, one should input the operating frequency *f* and material properties of the layers. Using this program, one can compute various dependencies, e.g., velocity versus viscosity of fluid or thickness of some layer.

**Figure 3:**
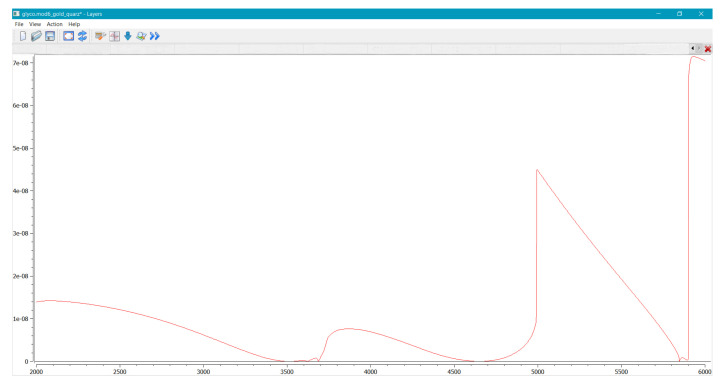
Snapshot of the program window showing the calculation of the fitting function.

Once the values of the parameters are set, the tensors $C^{GL}$ [$10^{9}$ N/m${}^{2}$] and $P^{GL}$ [$10^{-2}$ N· s/m${}^{2}$] are calculated and one can proceed with the calculation of the fitting function by choosing the program action “Calculate”. An example of the calculation of the fitting function is shown in Figure 3.

**Figure 4:**
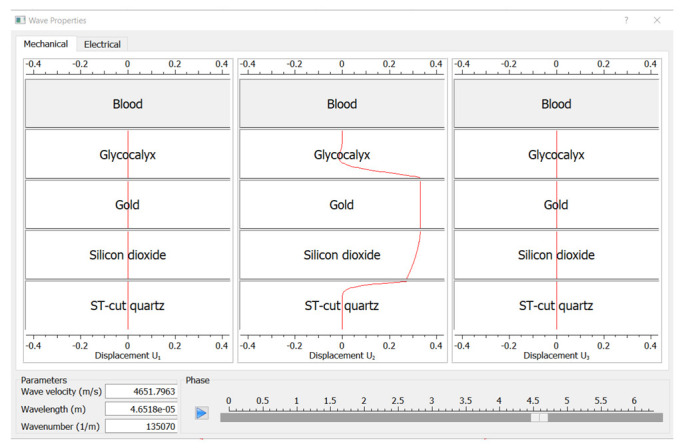
Snapshot of the program window showing the results on the properties of found waves.

Different roots of the fitting function correspond to different wave modes. To find out which root corresponds to pure shear waves, one should determine a sub-interval containing one particular root by zooming the root and use the action “Show the wave” to make sure that only $u_{2}$-displacements are present (see Figure 4). Once such a root is determined, one can calculate the corresponding wave velocity by choosing the action “Find the Minimum”.

## 3. Results

### 3.1. Geometrical and Physical Characteristics of Glycocalyx

Here, some overview about the data on glycocalyx geometrical and material properties will be given.

**Figure 5:**
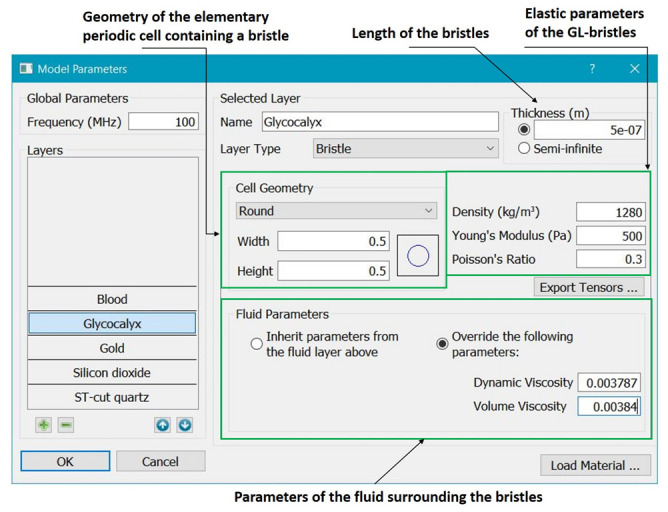
Snapshot of the program dialog for setting glycocalyx parameters.

The parameters of glycocalyx-like layer include the length of the villi (the thickness of glycocalyx), the elastic parameters (density, Young’s modulus, and Poisson’s ratio), as well as parameters of the surrounding blood (density, compressibility, and dynamic and volume viscosity). The geometric parameters (“width” and “height”) of the elementary periodic cell containing a single villi (see Figure 5) specify the relative villus size.

Some theoretical studies predicted the thickness of glycocalyx of about 0.5–1 μm [18,19], whereas the thickness up to 2.6 μm has been demonstrated in experimental works [20]. The atomic force microscopy [21] revealed the 380 nm-thick endothelial glycocalyx in human umbilical vein. In [22], the thickness of the glycocalyx layer in mammalian capillaries is estimated to be approximately of 0.4–0.5 μm. According to [23], the average thickness of glycocalyx is about 60–100 nm.

The Young modulus of glycocalyx has been estimated in [24] as 0.39 kPa. In [19], the value 0.7 ± 0.5 kPa has been determined. The paper [25] gives the Poisson ratio for hydrogels in the range of 0.25–0.4. The distance between individual villi is about 0.02 μm, the villi radius is in the range of 0.005–0.03 μm [26,27].

### 3.2. Numerical Simulations and Discussion

The values of material parameters used in the simulations are given in Table 1. The blood viscosity values were varied within a range of values observed in measurements for preterm and term neonates. The thicknesses of the guiding, shielding, and glycocalyx layers were 5 μm, 40 nm, and 350 nm, respectively. The operating frequency of the sensor of 100 MHz was chosen to provide pure shear wave modes.

**Figure 6:**
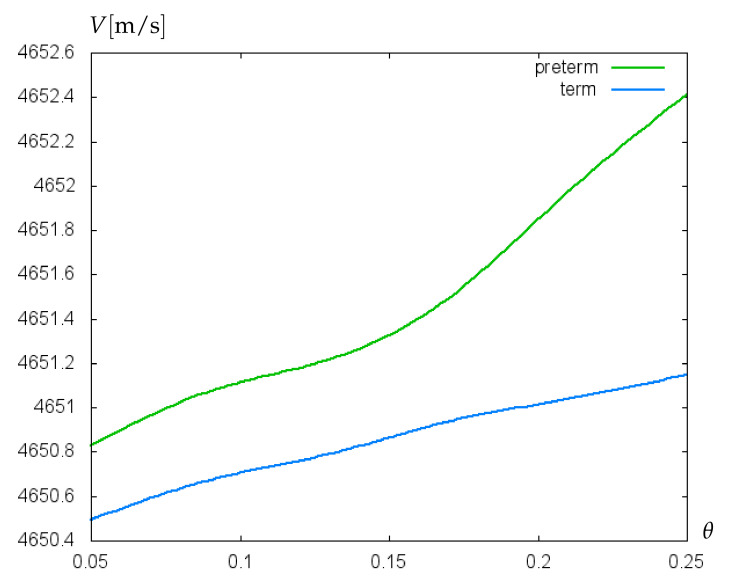
Velocity (m/s) of acoustic waves versus relative villus size, $\theta =\Sigma_{1}/\Sigma_{2}$ (cf. Figure 1b).

In Figure 6, the dependence of acoustic shear wave velocity on the relative villus size, $\theta =\Sigma_{1}/\Sigma_{2}$ is shown. In this simulation, the data on plasma viscosity for term and preterm infants are utilized. The green curve is related to a decreased value of the blood plasma dynamic viscosity equal to 0.00089 N·s/m${}^{2}$, which is typical for preterm infants [28]. The blue curve is computed for the blood plasma dynamic viscosity of 0.00104 N·s/m${}^{2}$ [28] corresponding to the mean blood plasma viscosity value for term neonates. Both curves are computed for the hematocrit (Ht) value of 40%, whereby the dynamic viscosity of the whole blood is calculated as *plasma viscosity/(1-Ht${}^{1/3}$)*. The increase of θ-values from 0.09 to 0.26 corresponds to the rise of villus radius from 0.0102 μm to 0.0136 μm. For both preterm and term neonates, an increase in relative villus size leads to an elevation of acoustic shear wave velocity. An increase in relative villus size enhances the rigidity of the surface layer material, which leads to a rise of the propagation speed of acoustic waves. However, in the case of preterm infants this effect is stronger because the lower blood plasma viscosity causes a lower liquid loading the wave propagation surface, which results in a higher wave propagation speed. A similar effect is noted in the paper [29].

**Figure 7:**
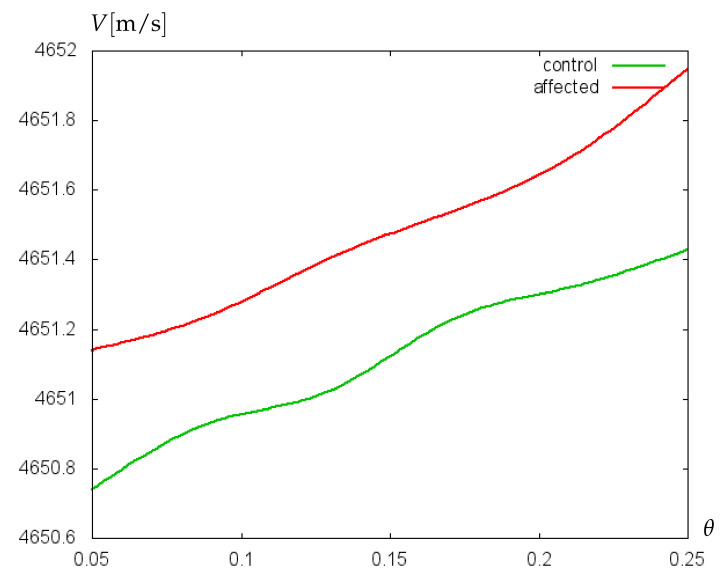
Velocity (m/s) of acoustic waves versus relative villus size, $\theta =\Sigma_{1}/\Sigma_{2}$, for experimentally collected data of extremely preterm infants.

**Figure 8:**
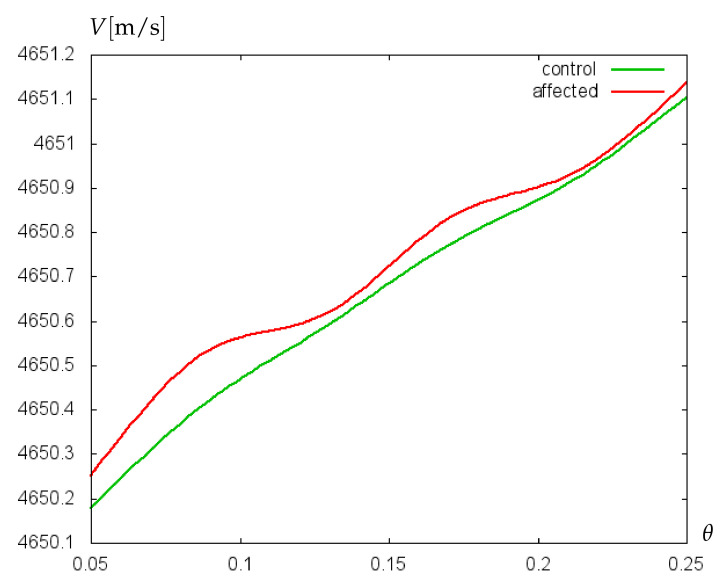
Velocity (m/s) of acoustic waves versus relative villus size, $\theta =\Sigma_{1}/\Sigma_{2}$, for experimentally collected data of very preterm infants.

Additionally, the sensitivity of the mathematical model to small variations of glycocalyx properties has been proved on experimental data collected retrospectively from clinical records of 229 preterm infants of two German clinics [30]. Patients were divided into two groups: without cerebral hemorrhage (control group) and with cerebral hemorrhage (affected group). Hematocrit values (Ht) from arterial and capillary blood samples were collected [31]. Neonates born at 23–26 weeks (extremely preterm) were considered separately from those born at 27–30 weeks (very preterm), and mean hematocrit values for every group were used to calculate the dynamic viscosity of blood. For control and affected groups of extremely preterm infants, the mean Ht-values were 44.2% and 42.2%, respectively. For very preterm infants, the mean Ht-values of control and affected groups were 46.7% and 45.8%, respectively. The results of computations with the mathematical model of biosensor are shown in Figure 7 and Figure 8. For extremely preterm infants (Figure 7), the difference between the control and affected group is more pronounced than for very preterm infants (Figure 8), due to a more essential difference between the mean Ht-values of control and affected groups. However, in both cases a good sensitivity of the algorithm to small variations of parameters has been established.

**Figure 9:**
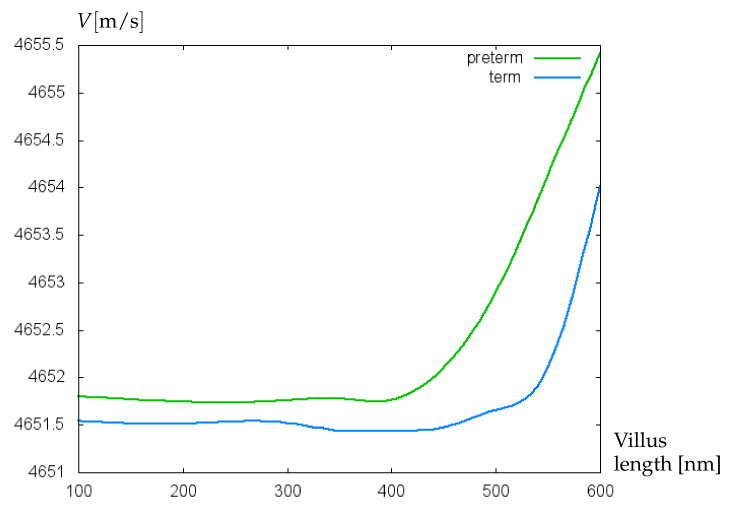
Velocity (m/s) of acoustic waves versus villus length (nm) for θ=0.3.

**Figure 10:**
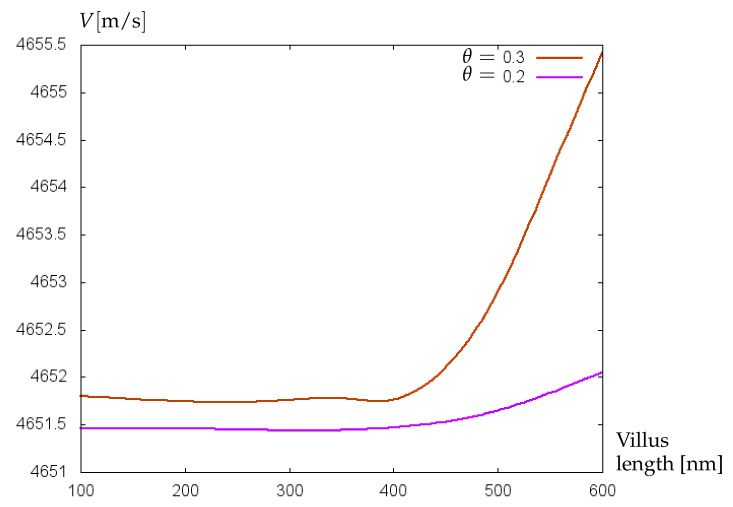
Velocity (m/s) of acoustic waves versus villus length (nm) for GL with θ=0.2 and θ=0.3 related to preterm infants.

In Figure 9, the effect of increasing villus length on the propagation velocity of acoustic shear waves is demonstrated. With the increase of the villus length from 100 μm to 400 μm the velocity of shear waves remains almost constant. With further extension of villus until 600 μm a noticeable increase in the velocity of shear waves appears for both preterm and term infants. It is known that a greater wave velocity corresponds to a more elastic material. One can speculate that glycocalyx with a longer villus length contributes to an increase of blood vessel elasticity. Note that this effect decreases with decreasing relative villus size, which is demonstrated in Figure 10, where the dependence of acoustic wave velocity on the villus length is shown for the relative villus size θ=0.2 (conform with the villus radius of 0.0102 μm) and θ=0.3 (conform with the villus radius of 0.0162 μm) and blood plasma viscosity value corresponding to preterm infants.

**Figure 11:**
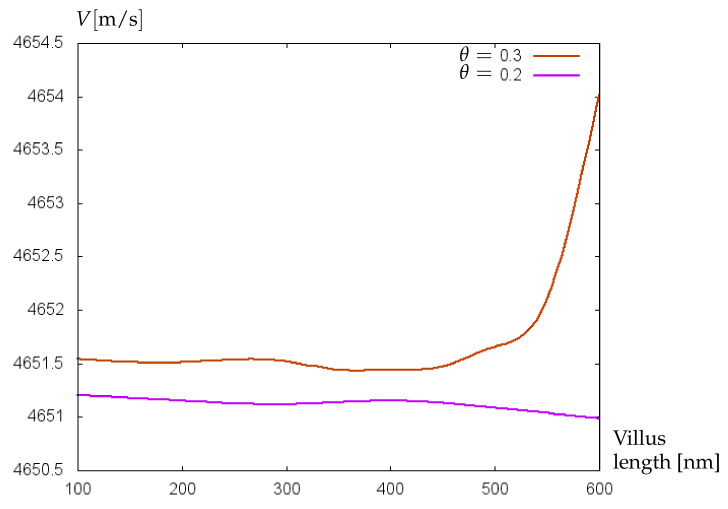
Velocity (m/s) of acoustic waves versus villus length (nm) for GL with θ=0.2 and θ=0.3 related to term infants.

An increase of blood plasma viscosity until the value corresponding to term infants, together with a decrease in relative villus size from θ=0.3 to θ=0.2, forces a stabilization of acoustic wave velocity with increasing villus length (see Figure 11). Since a decrease in relative villus size means the degradation of glycocalyx, the stabilization of acoustic wave velocity with a tendency to its lowering may be interpreted as diminishing elasticity of blood vessels, which is in accordance with the results of [32] substantiating the main contribution of glycocalyx to vessel wall elasticity.

The simulation results suggest that both the reduction of villi length and their diameter lead to a decrease in wave propagation velocity which may contribute to a decrease of vessel wall elasticity. By restoring a damaged glycocalyx, e.g., by means of infusion of glycocalyx components, the protective properties of vessel walls can be recovered [33].

**Figure 12:**
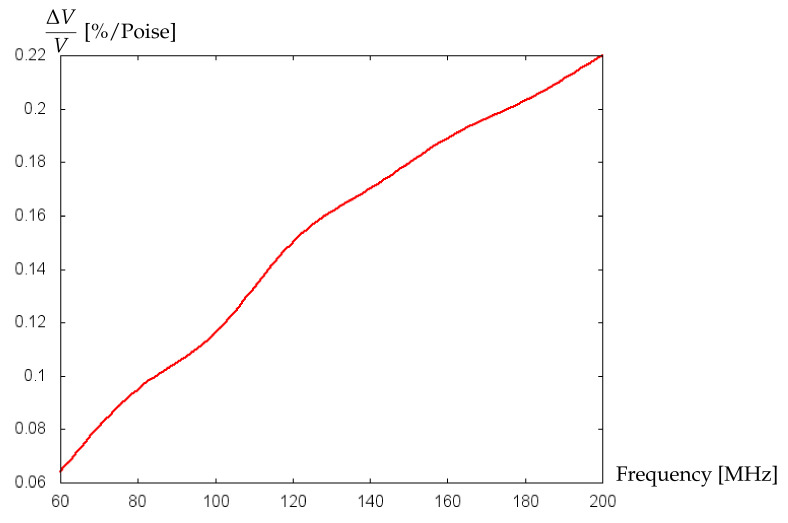
Change in the relative velocity of wave propagation per unit of the dynamic viscosity of blood (%/Poise) versus biosensor’s operating frequency (MHz).

Note that the sensitivity of the biosensor can be increased by increasing the operating frequency. Figure 12 shows the increase in the change of the relative velocity of wave propagation per unit of the dynamic viscosity of blood with the increase of operating frequency. However, one should take into account that the higher operating frequencies are associated with higher noise, which may hinder the achievement of high sensitivity. One can also try to optimize other parameters such as, e.g., substrate material, thickness of the guiding layer, etc., to achieve better sensitivity, which, certainly, deserves attention in future research.

## 4. Conclusions

The application of acoustic shear wave sensors seems to be very promising for the investigation of glycocalyx-like structures. Results of mathematical modeling suggest that high sensitivity of acoustic wave sensors to small changes of geometric and material properties of glycocalyx-like structures may allow better understanding the role of glycocalyx disturbances in prematurity.

## Figures and Tables

**Table 1:** Values of simulation parameters.

Layer	Material	Density [kg/m${}^{3}$]	Other Parameters
Fluid	Blood	$\rho^{f}$ = 1060	Dynamic viscosity, η = 0.0034 [N·s/m${}^{2}$]
			Volume viscosity, ζ = 0.00384 [N·s/m${}^{2}$]
			Compressibility, γ = 3.787e-10 [1/Pa]
Glycocalyx	Homogenized substance	$\rho^{GL}$ = 1280	Young’s modulus $E^{GL}$ = 500 [Pa]
			Poisson’s ratio $\nu^{GL}$ = 0.3
Shielding	Gold	$\rho^{g}$ = 19300	Young’s modulus *E* = 78 [GPa]
			Poisson’s ratio ν = 0.44
Guiding	SiO${}_{2}$	$\rho^{g}$ = 2200	Young’s modulus *E* = 72 [GPa]
			Poisson’s ratio ν = 0.17
Substrate	α-quarz	$\rho^{s}$ = 2650	Stiffness tensor *C* [$10^{9}$ N/m${}^{2}$]:
			$\begin{array}{cccccc} 86.74 & 6.99 & 11.91 & -17.91 & 0 & 0\\ 6.99 & 86.74 & 11.91 & 17.91 & 0 & 0\\ 11.91 & 11.91 & 107.2 & 0 & 0 & 0\\ -17.91 & 17.91 & 0 & 57.94 & 0 & 0\\ 0 & 0 & 0 & 0 & 57.94 & -17.91\\ 0 & 0 & 0 & 0 & -17.91 & 39.875 \end{array}$
			Piezoelectric tensor *e* [C/m${}^{2}$]:
			$\begin{array}{cccccc} 0.171 & -0.171 & 0 & -0.0407 & 0 & 0\\ 0 & 0 & 0 & 0 & 0.0407 & -0.171\\ 0 & 0 & 0 & 0 & 0 & 0 \end{array}$
			Dielectric tensor ε [$10^{-12}$ F/m]:
			$\begin{array}{ccc} 39.97 & 0 & 0\\ 0 & 39.97 & 0\\ 0 & 0 & 41.03 \end{array}$

## Data Availability

Medical data are available from the media and publications repository of the Technical University of Munich (mediaTUM) at the following link: https://mediatum.ub.tum.de/1521746 (accessed on 21 August 2022).
